# Symmetrical anterior–posterior partial fundoplication: technique and outcomes

**DOI:** 10.1007/s00464-026-12945-9

**Published:** 2026-06-19

**Authors:** Hideyuki Takeuchi, Andrés R. Latorre-Rodríguez, Lorenzo Cusmai, Arianna Vittori, Sumeet K. Mittal

**Affiliations:** 1 Norton Thoracic Institute, St. Joseph’s Hospital and Medical Center, 500 W Thomas Road, Suite 500, Phoenix, AZ 85013 USA; 2 Department of Surgery, The Jikei University School of Medicine, Tokyo, Japan; 3 Grupo de Investigación Clínica, Escuela de Medicina y Ciencias de La Salud, Universidad del Rosario, Bogotá. D.C, Colombia; 4 Division of General and Emergency Surgery, IRCCS Policlinico San Donato, University of Milan, Milan, Italy; 5 Department of Surgery, Oncology and Gastroenterology, School of Medicine, University of Padua, Padua, Italy; 6 Creighton University School of Medicine, Phoenix, AZ USA

**Keywords:** Partial fundoplication, Symmetrical anterior–posterior partial fundoplication, Gastroesophageal reflux disease, GERD, Outcomes, Patient-reported outcomes

## Abstract

**Background:**

Partial fundoplication is the most common surgical treatment for gastroesophageal reflux disease (GERD). Our preferred technique is a symmetrical anterior–posterior partial (300°) fundoplication created along the natural axis of the stomach, leaving the bare area on the esophagus along the lesser curvature. We assessed perioperative and long-term outcomes of this technique.

**Methods:**

We retrospectively reviewed records of all patients who underwent primary elective antireflux surgery by a single foregut surgeon from September 2016 to December 2024. Non-primary and emergency procedures and other techniques (such as Dor) were excluded. Perioperative complications, hiatal hernia recurrence, patient-reported outcomes (PROMs), and patient satisfaction were assessed for up to 7 years.

**Results:**

We identified 355 patients (254 [71.5%] women; median age, 64 years) who underwent primary antireflux surgery (laparoscopic [79.2%], robotic [20.6%], and conversion [0.3%]). The median operative time, estimated blood loss, and length of stay were 90 min (IQR 70–110), 25 mL (IQR 10–25), and 1 day (IQR 1–2). Severe complications (Clavien–Dindo grade ≥ III) occurred in 3 (0.8%) patients. There was no 90-day mortality. GERD health-related quality of life (GERD-HRQL) questionnaires and/or objective assessment using barium esophagram or upper GI endoscopy were completed by 222/355 (62.5%), 134/322 (41.6%), 56/267 (21.0%), 43/166 (25.9%), and 19/90 (21.1%) patients at 1, 2, 3, 5, and 7 years, respectively. The median total GERD-HRQL scores improved from 32 (IQR 13–46) preoperatively to 1 (IQR 0–4), 2 (IQR 0–7), 2 (IQR 0–12), 1 (IQR 0–6), and 5 (IQR 1–24) at 1, 2, 3, 5, and 7 years, respectively. Over 7 years, objective HH recurrence occurred in 22 patients (≥ 2 cm in 5 patients).

**Conclusions:**

Symmetrical anterior–posterior partial fundoplication is safe and effective, providing sustainable relief from GERD symptoms with high patient satisfaction reported up to 7 years. Interpretation of these promising results is limited by the small number of patients with extended follow-up.

**Supplementary Information:**

The online version contains supplementary material available at 10.1007/s00464-026-12945-9.

 Gastroesophageal reflux disease (GERD) results from chronic, repetitive pathological retrograde flow of gastric contents from a high-pressure intra-abdominal lumen (stomach) to a low-pressure intrathoracic lumen (esophagus) through an incompetent gastroesophageal junction (GEJ) barrier. The most common GERD symptoms are heartburn and regurgitation, and although antacids and acid suppression therapy provide symptomatic relief of pyrosis, the GEJ incompetence remains unaddressed. Indeed, other symptoms, especially those related to volume and less acidic refluxate, continue. Surgical (and more recently endoscopic) interventions aim to restore the antireflux barrier competence and provide definitive control of reflux [1, 2].

The optimal surgical technique for GERD remains a subject of ongoing debate. Several procedural variations have been described over the last four decades [3–5], and the most commonly used fundoplication configuration (i.e., fundic wrap) has changed over time (e.g., from an anterior–posterior wrap, popularized by Tom DeMeester in the open era to a greater curvature wrap introduced in the early laparoscopic era) [6]. At present, fundoplication techniques vary widely from complete to partial (2/3rd to 1/2 or even less). Despite this heterogeneity, high-volume centers have reported excellent long-term outcomes with up to 20–25 years of follow-up for minimally invasive fundoplication [7, 8]. Nevertheless, some outcomes are suboptimal with a significant proportion of patients reporting failure to control reflux and/or increased incidence of new undesirable side effects (i.e., new-onset postoperative dysphagia) [9]. If needed, most reoperative interventions are within 2–3 years [10, 11], indicating poor surgical technique in the first operation. In our opinion, these poor outcomes are the result of fundoplication techniques being an “art” rather than a widely reproducible surgical construct, thus relying on a prolonged mentorship and learning curve for the novice surgeon.

It is universally accepted that the fundoplication should be created using the fundus (the portion cephalad to the GEJ) of the stomach, and it should be placed around the distal esophagus rather than the proximal stomach and should lie tension free below the diaphragm. However, during the laparoscopic era, the technique was modified with fundoplication limbs being created along the greater curvature (not from the anterior and posterior fundus) and oriented anteriorly (likely due to ease of suturing in that location) as opposed to along the longitudinal axis of the stomach (lesser curvature).

The senior author (S.K.M.) has a standardized, reproducible technique to ensure adherence to the aforementioned principles. The fundoplication is created symmetrically along the natural axis of the stomach leaving the bare area on the esophagus along the lesser curvature. Over the years, we have made further changes while still adhering to the core principles listed above. Herein, we aimed to describe the symmetrical anterior–posterior partial fundoplication technique that provides a 300° fundoplication. This approach is specifically designed to create a reproducible antireflux barrier while minimizing side effects, such as dysphagia, by leaving the 9 o’clock (along lesser curvature) esophageal wall bare. Key steps include angle of His (AOH) accentuation and separation of the vagus nerves from the visceral wall to place the fundoplication limbs around the distal esophagus. We believe that the technique can be reproduced by adhering to these principles, improving the discordant outcomes reported in the literature. Therefore, we aimed to present and describe this novel fundoplication technique and to analyze its safety profile (perioperative morbidity) and outcomes (hiatal hernia [HH] recurrence and patient-reported outcomes [PROMs] using the GERD health-related quality of life [GERD-HRQL] questionnaire).

## Methods

### Study design and setting

We conducted an observational, retrospective study to review patients who underwent primary elective 300° symmetrical anterior–posterior partial fundoplication by the senior author (S.K.M.) from September 2016 to December 2024 at a high-volume tertiary center in Phoenix, AZ, USA. Patients who underwent redo procedures or non-elective procedures, who had advanced respiratory disease, or who received other types of fundoplication (e.g., Dor fundoplication) were excluded. The study was approved by the Institutional Review Board of St. Joseph’s Hospital and Medical Center, Phoenix, AZ, under the Norton Thoracic Institute Foregut Umbrella Protocol (PHXU-21–500-136–73-18). Moreover, The Surgical Technique Reporting Checklist and Standards (SUPER) was followed to report our results (Supplementary Material S1).

### Outcomes and data definitions

Data pertaining to demographics, procedure characteristics, and postoperative outcomes were queried from a prospectively maintained database of all benign foregut procedures. The *primary outcomes* were safety of the procedure (i.e., rates of intraoperative complications, intensive care unit [ICU] admissions, and early postoperative complications) and procedural effectiveness (i.e., HH recurrence and PROMs) with up to 7 years of postoperative follow-up. *Secondary outcomes* included operative time, estimated blood loss, and postoperative use of acid suppression therapy.

*Early postoperative complications* were defined as those occurring within 30 days of surgery, and the severity was graded using the Clavien–Dindo classification. *Objective HH recurrence* was defined as evidence of any part of the stomach above the diaphragm using upper gastrointestinal (GI) endoscopy or barium swallow studies at any postoperative time after surgery. Quality of life was assessed pre- and postoperatively using the total score from the validated, modified GERD-HRQL instrument, whereas postoperative patient satisfaction was assessed using a 10-point satisfaction scale (0 indicating the worst and 10 the best). Of note, for this study HH size was categorized based on the percentage of intrathoracic stomach noted at surgery, as described elsewhere [12].

### Surgical technique

All patients underwent an individualized preoperative evaluation by the surgeon, including contrast esophagram, upper GI endoscopy, high-resolution manometry, pH monitoring, and gastric emptying studies as clinically indicated. After review of the results and counseling, patients were offered antireflux procedures. Briefly, our novel approach involved a symmetrical, anterior–posterior partial fundoplication. This procedure encircles 300° of the esophageal circumference. The fundoplication limbs are positioned between the vagus nerves and the esophagus. More recently, we have incorporated placing an additional row of sutures to accentuate the AOH before rotating the fundoplication limbs.

Patients are positioned in an inverted Y-lithotomy position, with arms tucked in with the surgeon standing between the patient’s legs. The first assistant stands to the left of the patient. A standard five-port (four ports and one liver retractor) technique is used. The first port is placed above and left of the umbilicus and is used as the optics port. After obtaining pneumoperitoneum (10–15 mmHg) with CO_2_, the patient is positioned in a steep reverse Trendelenburg position (15°–25°). Under direct visualization, a 5-mm trocar is placed one fingerbreadth below the costal margin in the left anterior axillary line. A self-retaining liver retractor (Nathonson®) is placed just next to the xiphoid and held with a table-mounted holder. A 5-mm trocar is placed to the right of the midline in the epigastrium. A 12-mm trocar is placed in the left upper quadrant at about the mid-clavicular line just below the costal margin. For cases using the da Vinci Xi® system, four 8-mm ports (in addition to the liver retractor) are placed in a semi-linear arch at the level of the umbilicus, and an extra 12-mm port is placed in the right lower abdomen to facilitate passage of sutures.

The dissection starts with the division of the gastro-hepatic ligament and the phreno-esophageal membrane to enter the mediastinum. If there is a large hernia with a well-defined sac, then the assistant grabs the hernia sac within the mediastinum and turns it outward with downward traction; the sac is divided near the arch of the crus to enter into the avascular mediastinal plane. The hernia sac is completely dissected from the mediastinal structures, and the hiatus is circumferentially mobilized followed by mediastinal dissection with a target intra-abdominal esophageal length of at least 1.5 cm. A 1/4th-inch Penrose drain is placed to encircle the esophagus and vagus nerves to facilitate safe retraction to allow for high mediastinal dissection.

The sac–GEJ attachments to the crus desiccation posteriorly are divided. The top-most short gastric vessels are divided, although at times to get better access to the left crus pillar, they may be divided earlier. Importantly, both the anterior and posterior vagus nerves are identified and preserved. The attachments of the superior splenic pole to the diaphragm are divided, which allows for more robust sutures at the base of the left crus limb, and if mesh is placed, it allows the lateral edges of the mesh to lie between the spleen and the diaphragm.

The hiatal defect is then closed with a primary crural repair using interrupted, non-absorbable size 0 Ethibond™ sutures (figure of ‘eight’ and/or simple sutures). The repair is initiated posteriorly, with additional sutures placed anterolaterally (2 o’clock) and anteromedially (10 o’clock) as needed to ensure a tension-free closure while avoiding unnecessary anterior bowing of the esophagus as it traverses the hiatus [13]. If mesh is used, it is placed and secured at this time. Currently, we prefer an inverted C-shaped bioprosthetic mesh with left lateral extent tucked behind the spleen. Subsequently, the Penrose drain is removed. The hernia sac is then dissected off the greater curvature in a cephalad fashion and used as a handle to dissect the gastric fat pad to precisely identify the GEJ by the longitudinal esophageal muscle fibers merging with the gastric serosa: this is the AOH. The intra-abdominal length of the esophagus is measured from the arch of the crus to this point.

**Fig. 1:**
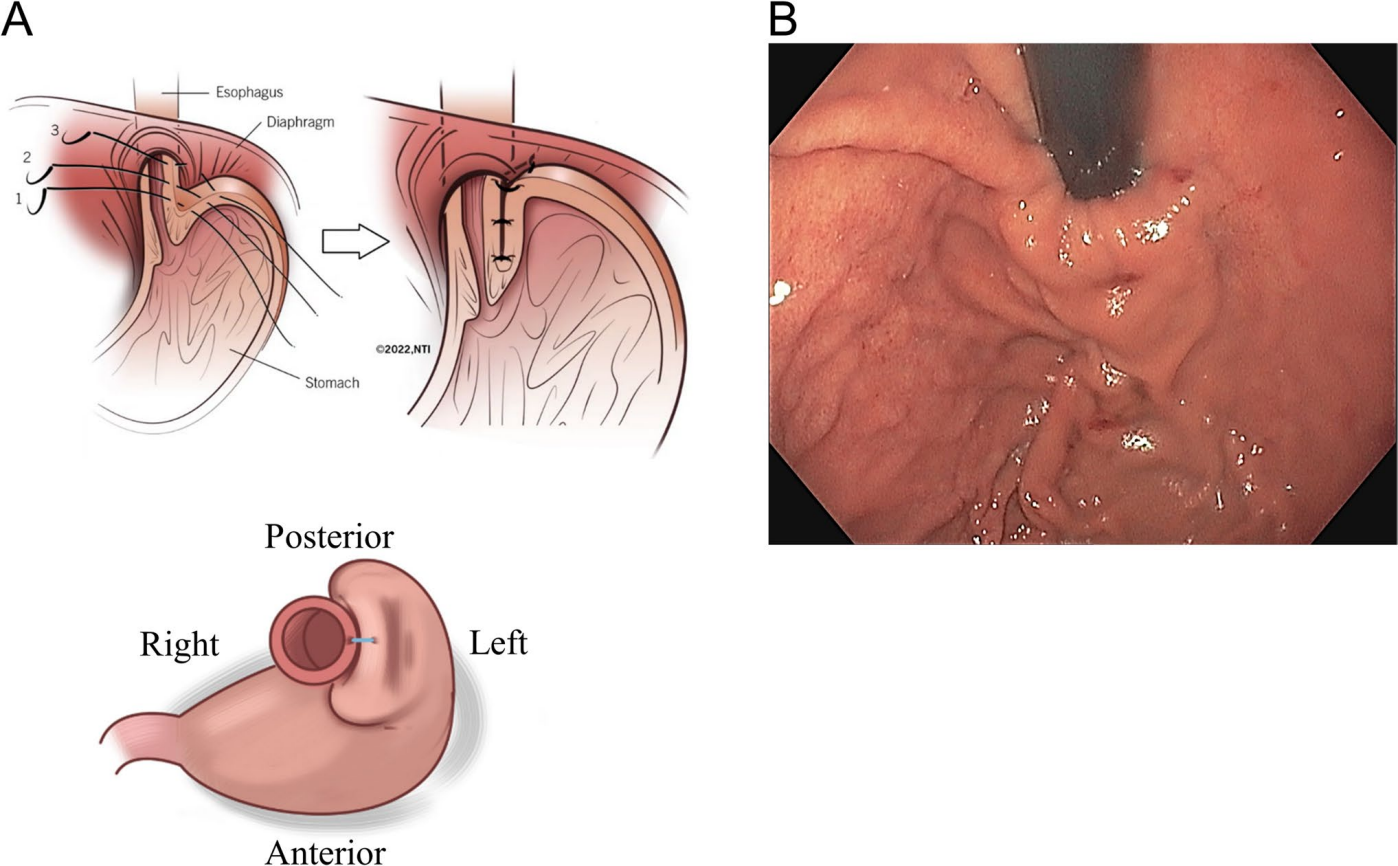
The angle of His (AOH) accentuation (**A**). The endoscopic image after the AOH fundoplication (**B**). Reproduced with permission from Norton Thoracic Institute

The gastric fat pad–sac is used as a handle to elevate the anterior vagus nerve and create a window between the anterior vagus and esophagus. The GEJ is retracted anteriorly by the assistant, and the left hand of the surgeon provides gentle cephalad and posterior traction behind the GEJ; this tents the posterior vagus while blunt dissection creates a window between the posterior vagus and esophagus. A Penrose drain is placed through the windows between the vagus nerves and esophagus. With the help of traction on the Penrose, the window between the vagus nerves and esophagus is widened to a distance of 2–3 cm with sharp energy aided dissection. Traditionally, the posterior fundoplication limb was marked with a stitch 5 cm away from the AOH and 2 cm behind the divided greater curvature prior to passing through the retro-esophageal window and then choosing a part of the anterior fundus for a shoe-shine maneuver until the correct point was identified by experience. Over the last 3 years, we have modified our technique slightly. We now accentuate the AOH as the first step after the hernia sac has been dissected off, and the window between the vagus nerves is created. This is performed by placing a series of three 2–0 Ethibond sutures to imbricate the fundus along the divided short gastrics onto the left-lateral aspect of the esophagus (3 o’clock position). The uppermost of these sutures also incorporates the arch of the left crus at the 3 o’clock position. This anchors 2 cm of intra-abdominal esophageal length and accentuates the AOH (Fig. 1A). AOH accentuation ensures proper positioning of the fundoplication limbs, effectively eliminating the need for a marking stitch and the shoe-shine maneuver. An endoscopic view after the AOH accentuation already shows a fairly robust antireflux barrier (Fig. 1B).

**Fig. 2:**
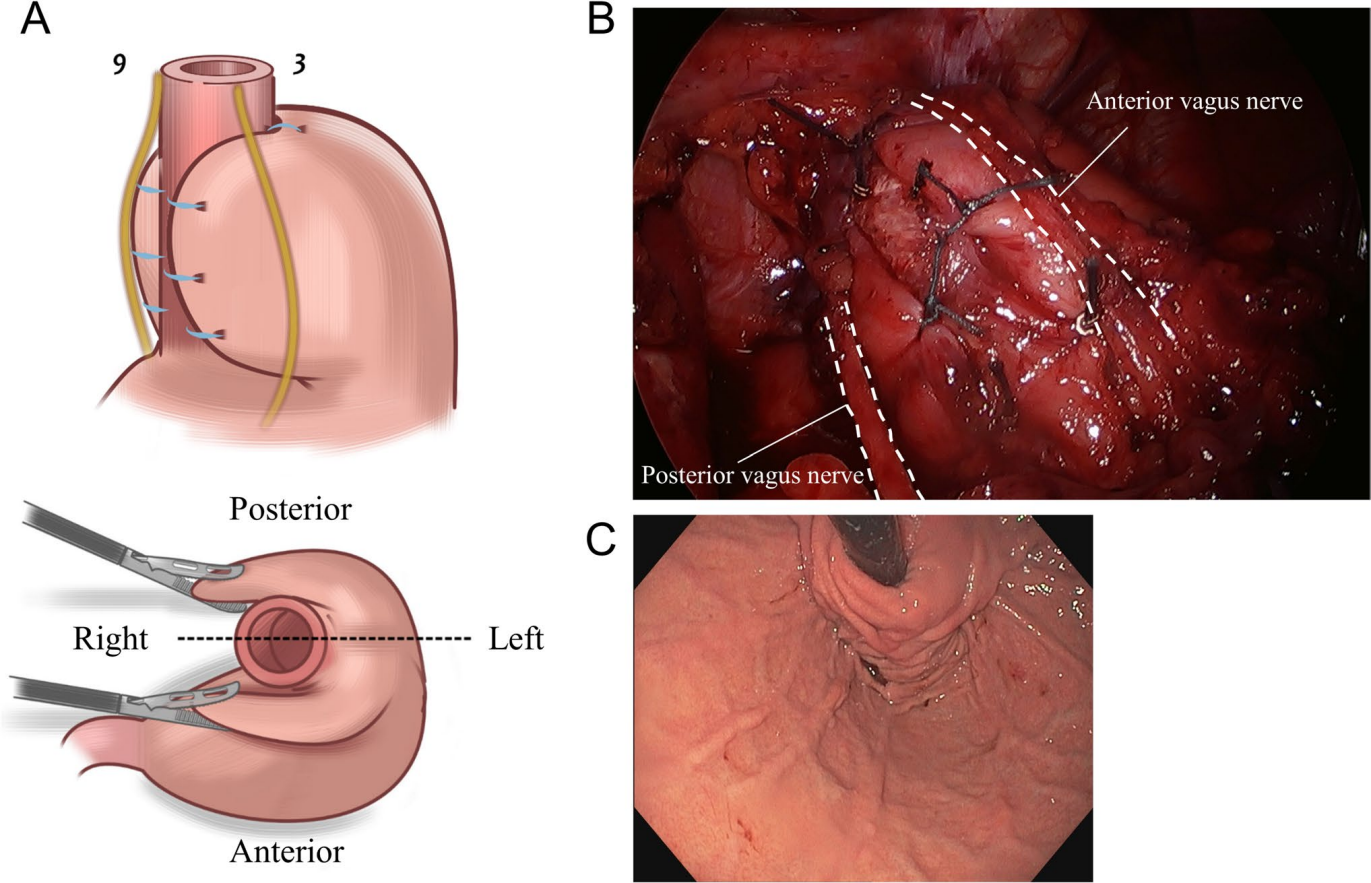
The anterior limb is brought over the anterior aspect of the esophagus (**A**). This provides a symmetrical anterior–posterior partial fundoplication (**B**) with the vagus nerves out of the wraps (white dashed line). The endoscopic view after the surgery (**C**). Reproduced with permission from Norton Thoracic Institute

Following this, the posterior limb of the fundus is passed through the retro-esophageal window and secured to the right-lateral aspect of the esophagus (approximately at the 8 o’clock position) with 3 interrupted sutures. The anterior limb is then brought over the anterior aspect of the esophagus and secured to the left-anterolateral esophagus (approximately at the 10 o’clock position), again with 3 interrupted sutures. This creates a partial anterior–posterior symmetrical fundoplication. The endoscopic view at the end shows a symmetric wrap (Fig. 2). For acute pain control, local anesthetic (0.25% bupivacaine hydrochloride without epinephrine [75 mg/30 mL]) is instilled over the left hemi-diaphragm before skin closure [14]. A detailed video describing the technique is available in Supplementary Material 2 (also available at https://youtu.be/kbuc2yYR9kw).

### Sampling and statistical analysis

All individuals meeting the inclusion criteria during the study period were included by convenience. Descriptive statistics were used to summarize the cohort characteristics and predefined outcomes. Continuous variables are reported as median with interquartile range [IQR], and categorical variables as count with proportion (%). Further, normality of the data distribution was assessed using the Shapiro–Wilk test and Q-Q plots. As for paired comparisons of pre- and postoperative data among the cohort (if both available), the Wilcoxon rank-sum test was used for continuous variables, whereas the McNemar test was used for categorical variables. Recurrence-free survival after HH repair was analyzed using Kaplan–Meier curves. The event of interest was recurrence of HH, and patients without recurrence were censored at the date of last postoperative follow-up or loss to follow-up. Statistical significance was set at *p* < 0.05. Version 4.5.1 of the R statistical software (R Foundation for Statistical Computing, Vienna, Austria) was used for statistical analyses.

## Results

### Baseline characteristics

Table 1 summarizes the cohort baseline characteristics. A total of 845 patients underwent benign foregut procedures during the study period, and 355 patients who underwent elective primary 300° symmetrical anterior–posterior partial fundoplication form the cohort of this study. The median age was 64 (IQR 54–71) years, 254 (71.5%) patients were female, and the median BMI was 28.8 (IQR 25.7–31.9) kg/m^2^. Nearly all (94.6%) patients were on acid suppression therapy preoperatively, mostly (90.9%) proton pump inhibitors (PPIs). The most common primary indications for surgery were reflux-related esophageal burden (267 [75.2%] patients), such as volume reflux and pyrosis, despite medical therapy. A preoperative HH was present in 343 (96.6%) patients, and the size distribution was as follows: small-HH: 161 (45.4%), moderate-HH: 77 (21.7%), large-HH: 64 (18.0%), and intrathoracic stomach: 41 (11.5%).

**Table 1:** Patient demographics and baseline characteristics

Variable	Cohort (*N* = 355)
*Demographics*	
Age, years	64 [54, 71]
Sex, female	254 (71.5)
BMI	28.8 [25.7, 31.9]
*Primary indication*	
Typical GERD/ volume reflux	267 (75.2)
Atypical GERD symptoms	17 (4.8)
Obstructive symptoms	51 (14.4)
Bleeding/anemia	38 (10.7)
Asymptomatic PEH	1 (0.3)
*Hiatal hernia size*	
None	12 ( 3.4)
Small	161 (45.4)
Moderate	77 (21.7)
Large	64 (18.0)
Intrathoracic stomach	41 (11.5)

Data presented as median [IQR] or no (%). *BMI* body mass index, *GERD* gastroesophageal reflux disease, *PEH* paraesophageal hiatal hernia; *HH* hiatal hernia

### Operative data and surgical details

Table 2 presents the surgical details and intraoperative outcomes. Most of the procedures were completed laparoscopically (*n* = 281, 79.2%), followed by robotic-assisted (*n* = 73, 20.6%), with 1 case (0.3%) requiring conversion to open surgery. The median intra-abdominal esophageal length achieved was 2 cm (IQR 1.5–2.5), and a bioprosthetic mesh (GORE® BIO-A® or Phasix-ST) was utilized to reinforce the crural repair in 82 (23.1%) cases. The median operative time was 90 min (IQR 70–110), the estimated blood loss was 25 mL [IQR 10–25], and the median length of hospital stay was 1 day (IQR 1–2).

**Table 2:** Surgical characteristics and intraoperative details

Variable	Cohort (*N* = 355)
Operative time, min	90 [70–110]
Blood loss, mL	25 [10–25]
*Surgical approach*	
Laparoscopic	281 (79.2)
Robotic	73 (20.6)
Conversion to open	1 (0.3)
Use of mesh	82 (23.1)
Esophageal abdominal length, cm	2 [1.5–2.5]
Anterior vagus nerve out of wrap†	323 (91.0)
Posterior vagus nerve out of wrap†	332 (93.5)
*Intraoperative complication*	15 (4.2)
Anterior vagus nerve injury	3 (0.8)
Perforation of EGJ	1 (0.3)
Perforation of stomach	1 (0.3)
Splenic bleeding	4 (1.1)
Other bleeding (liver, short gastric artery, etc.)	6 (1.7)
Hospital stay, days	1 [1, 2]
ICU admission	7 (2.0)

Data presented as median [IQR] or no (%). *EGJ* esophagogastric junction, *ICU* intensive care unit

^†^Indication of whether separating the anterior and posterior vagus nerve from the esophagus wall

Intraoperative complications were noted in 15 (4.2%) patients. Anterior vagus nerve injury (i.e., division) occurred in 3 (0.8%) patients, splenic bleeding in 4 (1.1%) patients, and bleeding from other sources (e.g., liver or short gastric artery) in 6 (1.7%) patients; however, significant bleeding (> 300 mL) did not occur in any patients. Further, 7 (2.0%) patients required ICU admission for observation related to preexisting medical conditions. Of these, 1 (0.3%) patient had a postoperative hemorrhage requiring surgical reintervention.

### Early postoperative outcomes

Table 3 summarizes the occurrence and nature of early postoperative complications. Overall, postoperative complications were observed in 28 (7.9%) patients. The most frequently observed complication was atrial fibrillation (*n* = 5, 1.4%), followed by paralytic ileus and urinary retention (each *n* = 3, 0.8%). Severe complications (Clavien–Dindo grade ≥ III) occurred in 3 (0.8%) patients, 1 with postoperative hemorrhage necessitating a return to the operating room, 1 with acute gastric distention and the need for a percutaneous endoscopic gastrostomy (PEG) placement, and 1 with early dislodgement of the PEG tube placed at the time of surgery resulting in the formation of an abdominal wall abscess requiring surgical debridement. There was no perioperative or 90-day mortality.

**Table 3:** Postoperative morbidity

Variable	Cohort (*N* = 355)
*Any postoperative complication*, (%)	28 (7.9)
Atrial fibrillation	5 (1.4)
Persistent paralytic ileus	3 (0.8)
Urinary retention	3 (0.8)
Abdominal wall abscess	1 (0.3)
Anemia (Hb < 10 g/dL)	1 (0.3)
Gastric distention	1 (0.3)
Persistent pneumothorax	1 (0.3)
Postoperative bleeding	1 (0.3)
Viscus injury	1 (0.3)
Other minor complications	11 (3.1)
*Clavien–Dindo classification*, (%)	
Grade I	12 (3.4)
Grade II	13 (3.7)
Grade IIIb	2 (0.6)
Grade IVb	1 (0.3)

Data presented as no (%)

### Surgical outcomes

Table 4 presents outcomes including HH recurrence and acid suppression therapy requirement along with pre- and postoperative quality-of-life measurements. Follow-up including the GERD-HRQL instrument and/or objective assessment using barium esophagram or upper GI endoscopy was completed at scheduled follow-up appointments by 222/355 (62.5%) patients at 1 year, 134/322 (41.6%) at 2 years, 56/267 (21.0%) at 3 years, 43/166 (25.9%) at 5 years, and 19/90 (21.1%) at 7 years.

**Table 4:** Mid-term outcomes including patient-reported quality of life before and after antireflux surgery

	Preoperative		Year 1	Year 2		Year 3		Year 5		Year 7	
Patients eligible for follow-up	355		355	322		267		166		90	
*Medical therapy*											
Acid suppression therapy use^a^	228/241 (94.6)		10/155 (6.5)**⁑**	18/113 (15.9)**⁑**		12/76 (15.8)**⁑**		15/67 (22.4)**⁑**		6/21 (28.6)	
PPI use	219/241 (90.9)		6/155 (3.9)**⁑**	12/113 (10.6)**⁑**		6/76 (7.9)**⁑**		9/67 (13.4)**⁑**		5/21 (23.8)	
H2-receptor blockers	33/241 (13.7)		4/155 (2.6)**⁑**	7/113 (6.2)		7/76 (9.2)		6/67 (9.0)		1/21 (4.8)	
*Patient-reported outcomes*											
Completed GERD-HRQL instrument^a^	221/355 (62.3)		140/355 (39.4)	105/322 (32.6)		69/267 (25.8)		63/166 (38.0)		19/90 (21.1)	
Total GERD-HRQL score, median	32 [13–46]		1 [0–4]**⁑**	2 [0–7]**⁑**		2 [0–12]**⁑**		1 [0–6]**⁑**		5 [1–24]	
Heartburn subscore, median	15 [8–22]		0 [0–2]**⁑**	0 [0–3]**⁑**		0 [0–5]**⁑**		0 [0–4]**⁑**		5 [0–10]	
Dysphagia subscore, median	2 [0–4]		0 [0–1.5]⁑	0 [0–2]*		0 [0–2]*		0 [0–1]**⁑**		0 [0–2]	
Significant dysphagia (score ≥ 5)	54/221 (24.4)		10/140 (7.1)**⁑**	12/105 (11.4)		10/69 (14.5)		2/63 (3.2)		2/19 (10.5)	
Regurgitation subscore, median	13 [3–21]		0 [0–0]**⁑**	0 [0–1]**⁑**		0 [0–4]**⁑**		0 [0–1]**⁑**		0 [0–11.0]	
Completed patient-reported satisfaction survey^a^	–		108/355 (30.4)	92/322 (28.6)		56/267 (21.0)		58/166 (35.0)		19/90 (21.1)	
High satisfaction (≥ 8/10)	–		94/108 (87.0)	73/92 (79.3)		43/56 (76.8)		47/58 (81.0)		14/19 (73.7)	

Continuous variables were compared between preoperative and each follow-up timepoint using the Wilcoxon rank-sum test; categorical variables were compared using the McNemar test

Data presented as median [IQR] or no (%). *CT* computed tomography, *GERD-HRQL* gastroesophageal reflux disease health-related quality of life

^*^p-value: < 0.05, ⁑ *p*-value: < 0.001

^a^Adjusted to available data

**Fig. 3:**
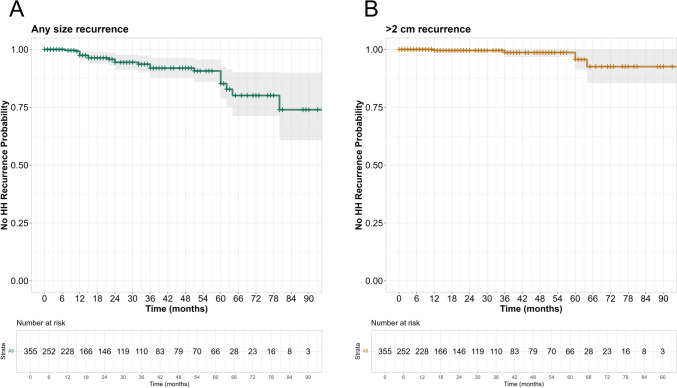
Kaplan–Meier curve for recurrence-free survival after antireflux surgery (based on the last clinical follow-up) is plotted for the entire cohort

Over the 7-year follow-up, objective HH recurrence of any size was observed in 22 patients, of whom 5 (1.4%) had a recurrent HH greater than 2 cm (Fig. 3). In this cohort, 3 patients required reoperative procedures: 1 for recurrent reflux 66 months after primary surgery, 2 for dysphagia, 1 for mesh-related hiatus stenosis at 12 months, and 1 with a recurrent paraesophageal hernia 20 months after the primary surgery.

**Fig. 4:**
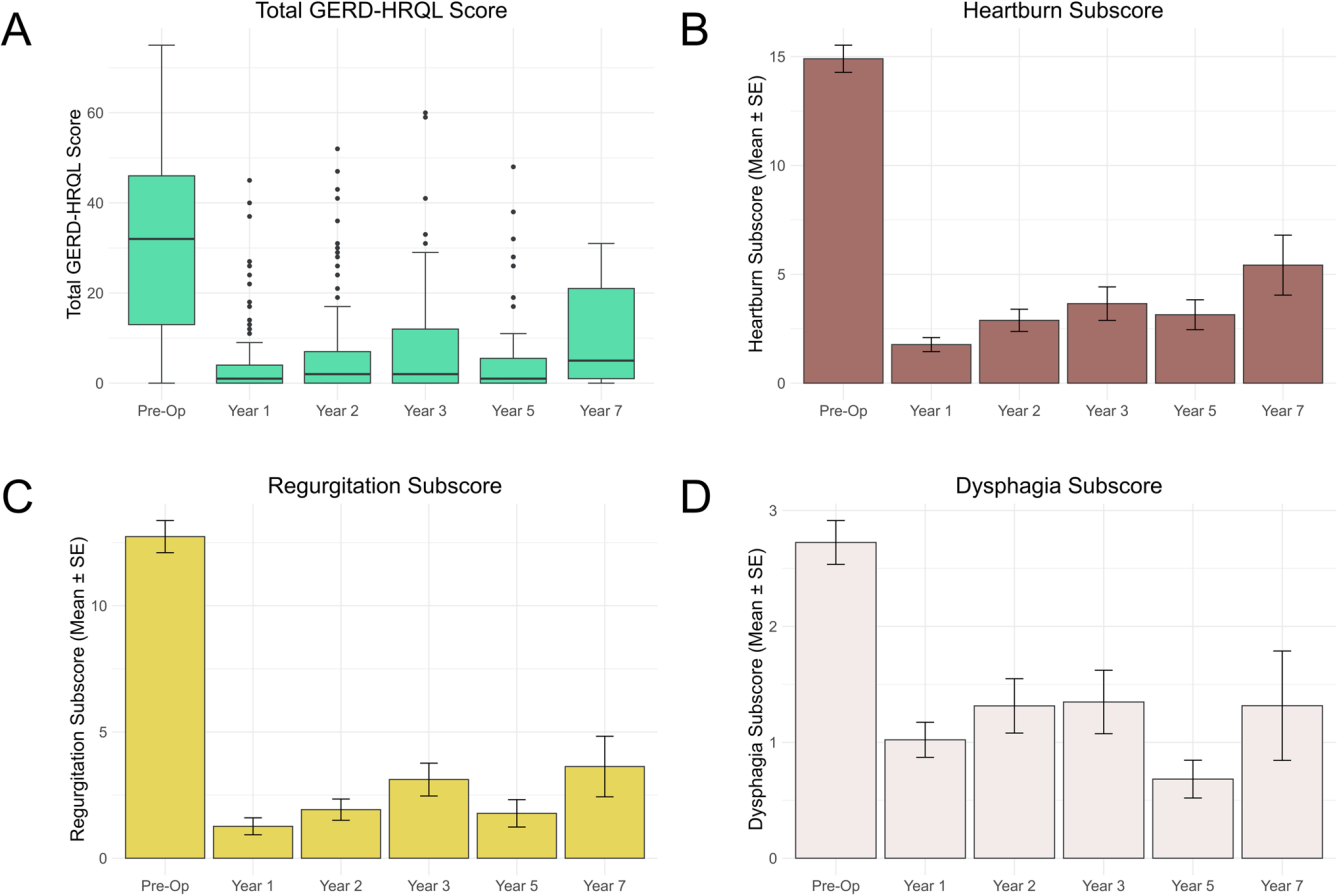
Total gastroesophageal reflux disease health-related quality of life (GERD-HRQL) score (**A**) and GERD-HRQL subcomponent scores: heartburn (**B**), regurgitation (**C**), and dysphagia (**D**)

 The proportion of patients taking acid suppression therapy decreased substantially from 228/241 (94.6%) preoperatively to 10/155 (6.5%) at 1 year (*p* < 0.001), 18/113 (15.9%) at 2 years (*p* < 0.001), 12/76 (15.8%) at 3 years, 15/67 (22.4%) at 5 years (*p* < 0.001), and 6/21 (28.6%) at 7 years. Furthermore, the median total GERD-HRQL score for the cohort improved from 32 (IQR 13–46) preoperatively to 1 (IQR 0–4; *p* < 0.001), 2 (IQR 0–7; *p* < 0.001), 2 (IQR 0–12; *p* < 0.001), 1 (IQR 0–6; *p* < 0 0.001), and 5 (IQR 1–24) at 1, 2, 3, 5, and 7 years postoperatively, respectively. This improvement was driven by lower scores across all major symptom domains (Fig. 4). Notably, the majority of patients with significant postoperative dysphagia (subcomponent score ≥ 5) had also reported significant preoperative dysphagia.

Among those who answered the patient satisfaction scale in the follow-up instrument, 94/108 (87.0%), 73/92 (79.3%), 43/56 (76.8%), 47/58 (81.0%), and 14/19 (73.7%) reported very high satisfaction (a score of ≥ 8) at 1, 2, 3, 5, and 7 years, respectively.

## Discussion

It is widely accepted that fundoplication restores the antireflux barrier components by repairing the hiatal hernia, reinforcing the lower esophageal sphincter function and reconstructing the gastroesophageal flap valve mechanism through the AOH accentuation [15–17]. There is clinical consensus that the wrap (fundus) should be around the distal esophagus and below the diaphragm [18–20]. A twisted wrap results when parts of the fundus too distant from the GEJ are mistakenly chosen; whereas a misplaced/slipped wrap is when the fundoplication is placed around the proximal stomach (as opposed to the distal esophagus). A twisted or misplaced (i.e., on the proximal stomach) fundoplication results in poor outcomes [21–23], and a slipped fundoplication is a common finding in patients with recurrent symptoms. Latorre et al. [24] reported that 40.5% of patients undergoing reoperative surgery were found to have a slipped fundoplication. Our novel technique is unique as it mandates the creation of a symmetrical anterior–posterior partial fundoplication around the distal esophagus obviating a twisted or a misplaced wrap. Further, it appears to be safe and effective, providing excellent patient-reported outcomes.

Variability in surgical outcomes often depends on the institution and surgeon’s experience because operative techniques are not always standardized. Standardization plays a crucial role in avoiding incorrect fundoplication configurations, such as a misplaced fundoplication or twisting of the fundus. Surgeons may inadvertently grasp the most accessible point on the gastric wall, particularly for the posterior limb, rather than the anatomically correct location for fundoplication. Our standardized symmetrical anterior posterior partial fundoplication provides two key advantages. First, it creates a symmetric untwisted wrap as the steps of the procedure mandate using the correct fundic landmarks. Reardon et al. [25] have earlier reported that most Nissen fundoplications were incorrect due to the wrap being twisted. They recommend placing a marking stitch to pull the correct portion of the posterior limb followed by the shoe-shine maneuver. Second, our standardized technique reconstructs an acute AOH by suturing the greater curvature of the fundus to the esophagus at the 3 o’clock orientation, which is independently associated with reduced reflux. In our modification of the technique, the initial AOH accentuation stitches are placed as the first step, automatically positioning the fundic limbs and precluding the requirement of a marking stitch or the shoe-shine maneuver. A wider AOH has been shown to correlate with higher rates of GERD; however, after this angle is reconstructed through fundoplication, a flap valve, which functions as a natural physiologic and anatomic barrier, becomes endoscopically visible as a good mucosal fold [17]. In fact, Nguyen’s “Omega fundoplication” endoscopic perspective validates the anatomical rationale of a partial fundoplication design [20]. In this study, the reason for our consistent and excellent outcomes lies in the anatomical rationality of our technique. While current fundoplication techniques often yield variable outcomes [26, 27], likely due to technique heterogeneity, our protocol ensures reproducibility through a standardized step-wise creation of the fundoplication.

Another important consideration is the separation of the vagus nerves from the esophagus. Although the fundoplication can be created around the vagus nerves (without separating them from the esophagus to create the windows for fundoplication limbs), we believe that separating the vagus nerves allows for the removal of hernia sac remnants, the gastric fat pad, adventitial tissue, and inflammatory fibrotic debris around the esophagus, which leads to the correct identification of the GEJ. This also ensures that dissection is consistently performed on the true esophagus with reliable anatomical guidance and that sutures secure the fundoplication to the esophagus and not the hernia sac adventitia. Of further advantage, these steps ensure that the fundoplication is created around the esophagus (not misplaced onto the proximal stomach) in the first place and cannot ‘slip’ on the proximal stomach in the future. Using a gentle and precise standardized technique, we can separate the anterior and posterior vagus nerves safely in nearly all patients. Separation of the vagus nerves is not particularly challenging and provides substantial anatomical and procedural benefit. In a review study, Rijin et al. [28] reported that the prevalence of vagus nerve injury after antireflux surgery appeared to be 10% to 42%; however, in our study, we observed only 3 (0.8%) cases of anterior vagus nerve injury. None of these patients had symptoms of delayed gastric emptying.

One of the feared complications of fundoplication has been postoperative dysphagia—most commonly associated with a twisted or slipped fundoplication. In our study, 7.1% (10/140) of patients reported significant dysphagia (score ≥ 5) at 1 year postoperatively (most of them had reported significant preoperative dysphagia). This is much lower than the 14.7% (5/34) reported for Nissen fundoplication and comparable to the 2.6% (1/38) reported for Lind fundoplication at 1 year in a randomized controlled trial by Khan et al. [29]. Consistent with the literature, the strongest predictor of postoperative dysphagia in our cohort was preoperative dysphagia (analysis not shown).

Although we did not evaluate the number of patients who had significant bloating, a 300° wrap provides a balanced trade-off between reflux control and new-onset postoperative dysphagia and gas-bloat symptoms [30]. The improvement in GERD-HRQL scores and high patient satisfaction provide further evidence of its effectiveness. The number of patients taking acid suppression medications at any time after surgery is the only recurrence data available, and the reason for the resumption of medications is not documented.

In our study, the cumulative recurrence-free hiatal hernia (> 2 cm) rate was 92.6% over 90 months of follow-up. Most recurrences occurred in the late follow-up period (> 60 months). This is significantly better than the generally reported recurrence rates in the range of 25% to 42% [31]. Armijo et al. [32] reported an even higher rate, with 60% (30/50) of patients developing recurrent hiatal hernia (> 2 cm) during their study period. Although direct comparison is limited by the heterogeneity of follow-up durations across studies, ranging from 6 months to 10 years, our recurrence rate compares favorably with these published data, indicating that our standardized surgical technique and rationale contribute to improved anatomical outcomes.

Patient-reported outcome measures are now widely accepted as essential tools for evaluating surgical efficacy and quality from the patient’s perspective [33, 34]. Analysis of our cohorts demonstrated statistically significant and sustained improvements in GERD-HRQL scores and its subcomponent scores (heartburn, regurgitation, and dysphagia) as well as high patient satisfaction, consistent with findings reported by other authors [35, 36].

Our study has some limitations. First, this is a novel technique, and the nature of the study is descriptive as there was no direct comparator (i.e., other fundoplication techniques) nor randomization; thus, external validation is required. Second, the follow-up horizon is still somewhat limited (up to 7 years), and analysis of long-term outcomes with a larger cohort is necessary. Third, this is a single-center study with a single experienced foregut surgeon, and we do not have any data on the learning curve. Indeed, this aspect also needs to be studied in the future. Fourth, data on pH-monitoring metrics and particularly postoperative objective control of distal esophageal exposure were not available. Fifth, there is a limited proportion of patients with follow-up data; however, this is a consistent problem noted in the literature where response rates in similar studies are in the range of 49% to 54% [37, 38]. Finally, the impact of potential confounding factors such as surgical approach (i.e., laparoscopic or robotic) or the use of mesh reinforcement was not controlled in the analysis as the scope of the study was to present an overall description of the surgical technique.

## Conclusion

We introduce our standardized symMetrIcal anTerior–posTerior pArtiaL (MITTAL) fundoplication with AOH accentuation. It is associated with excellent objective and PROM outcomes along with very low perioperative morbidity.

## Supplementary Information

Below is the link to the electronic supplementary material.

Supplementary file1 (DOCX 1907 KB)
